# Anti-alpha-amino-3-hydroxy-5-methyl-4-isoxazolepropionic acid receptor encephalitis developed after ovarian cancer cytoreduction surgery: a case report and literature review

**DOI:** 10.1186/s12905-023-02636-1

**Published:** 2023-09-21

**Authors:** Yue Huang, Muke Zhou, Jing Zhou, Bo Wu, Xi Yang, Wenjiao Min, Zhengyu Li

**Affiliations:** 1 Department of Gynecology and Obstetrics, West China Second University Hospital, Sichuan University, Chengdu, 610041 People’s Republic of China; 2 Key Laboratory of Birth Defects and Related Diseases of Women and Children (Sichuan University), Ministry of Education, Chengdu, 610041 People’s Republic of China; 3 Department of Neurology, West China Hospital, Sichuan University, Chengdu, 610041 People’s Republic of China; 4 Department of Gynecology and Obstetrics, Chengdu Fifth People’s Hospital, Chengdu, 610041 People’s Republic of China; 5 Department of Anesthesiology, West China Hospital, Sichuan university, Chengdu, 610041 People’s Republic of China; 6 Department of Psychosomatic Medicine, Sichuan Academy of Medical Science & Sichuan Provincial People’s Hospital, Chengdu, 610041 People’s Republic of China

**Keywords:** Anti-AMPAR encephalitis, Paraneoplastic syndrome, Ovarian cancer, Cytoreduction surgery, Case report

## Abstract

**Background:**

Anti-alpha-amino-3-hydroxy-5-methyl-4-isoxazolepropionic acid receptor (AMPAR) encephalitis, a rare subtype of autoimmune encephalitis (AE), is often found associated with tumors such as thymoma, lung cancer, ovarian tumors, and breast cancer, and the tumors were generally detected during the screening process after the encephalitis initiated. The tumor is considered a trigger of AE, but the mechanism remains unclear.

**Case Presentation:**

A 53-year-old woman presented short-term memory loss two days after the primary cytoreduction for high-grade serous ovarian cancer (HGSOC, FIGO stage IC3). Cell-based assay found AMPAR CluA2 IgG positive in both serum (1:3.2) and cerebrospinal fluid (1:32). Moreover, mild AMPAR GluA1 and strong GluA2 expressions were also found positive in the paraffin sections of ovarian tumor tissue, indicating the ovarian cytoreduction surgery might stimulate the release of receptor antigens into the circulation system. The patient’s condition deteriorated within two weeks, developing consciousness and autonomic dysfunction, leading to ICU admission. With oral steroids, intravenous immunoglobulin, plasmapheresis, and rituximab treatment, the patient’s consciousness markedly improved after three months.

**Conclusion:**

We presented the first case of anti-AMPAR encephalitis developed right after the primary cytoreduction of a patient with HGSOC and retrieved paraneoplastic anti-AMPAR encephalitis cases (n = 66). Gynecologists should pay attention to patients who develop cognitive dysfunction or psychiatric symptoms shortly after the ovarian tumor resection and always include AE in the differentiation diagnosis.

**Supplementary Information:**

The online version contains supplementary material available at 10.1186/s12905-023-02636-1.

## Background

Anti-alpha-amino-3-hydroxy-5-methyl-4-isoxazolepropionic acid receptor (AMPAR) encephalitis was first described and identified by Lai et al. in ten patients with limbic encephalitis (LE) in 2009 [1]. Most paraneoplastic anti-AMPAR encephalitis patients present acute or subacute onset of short-term memory loss and/or psychiatric symptoms, with the concurrent or recurring tumors (lung cancer, thymoma, breast cancer, etc.) commonly detected in the following screening process [2, 3]. Anti-AMPAR encephalitis associated with ovarian cancer is rare. Here, we report a patient who developed encephalitis symptoms right after the primary cytoreduction for high-grade serous ovarian cancer (HGSOC).

## Case presentation

**Fig. 1:**
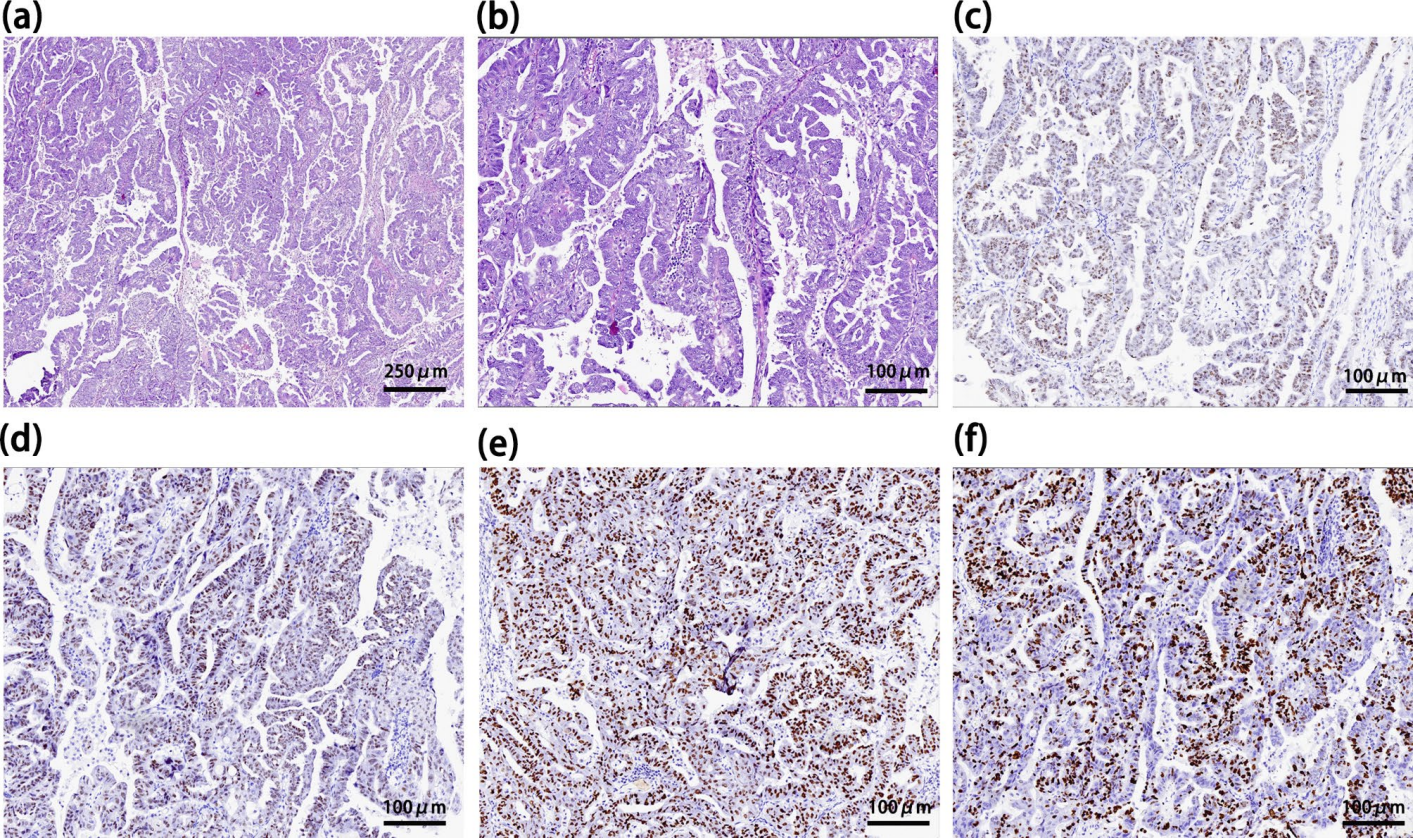
Ovarian pathology examination. Immunohistochemistry stain for **(a)** HE (40×), **(b)** HE (100×), **(c)** PAX8(100×), **(d)** WT1(100×), **(e)** p53(100×), and **(f)** Ki67 (100×), confirming that the histopathological type is high-grade serous ovarian cancer (HGSOC).

**Fig. 2:**
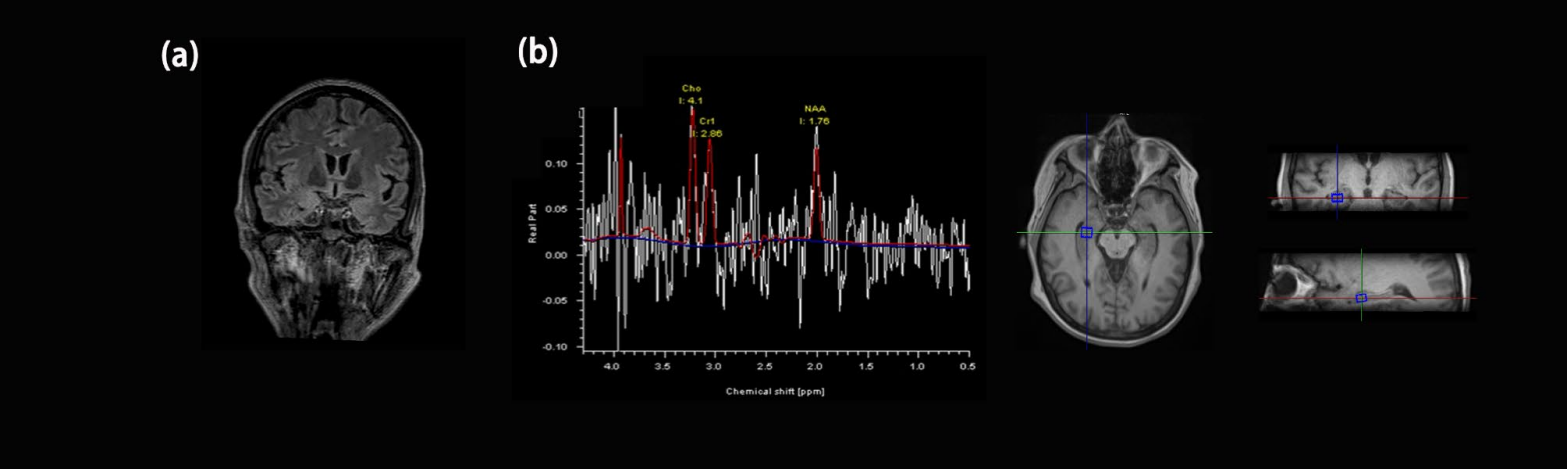
**(a)** Brain magnetic resonance image (MRI) showed coronal T2-FLAIR image revealed bilateral hippocampus volume decreased and **(b)** Representative spectra (echo time, 135 milliseconds) of right hippocampus region showed diminished NAA/(Cho + Cr) ratio

**Fig. 3:**
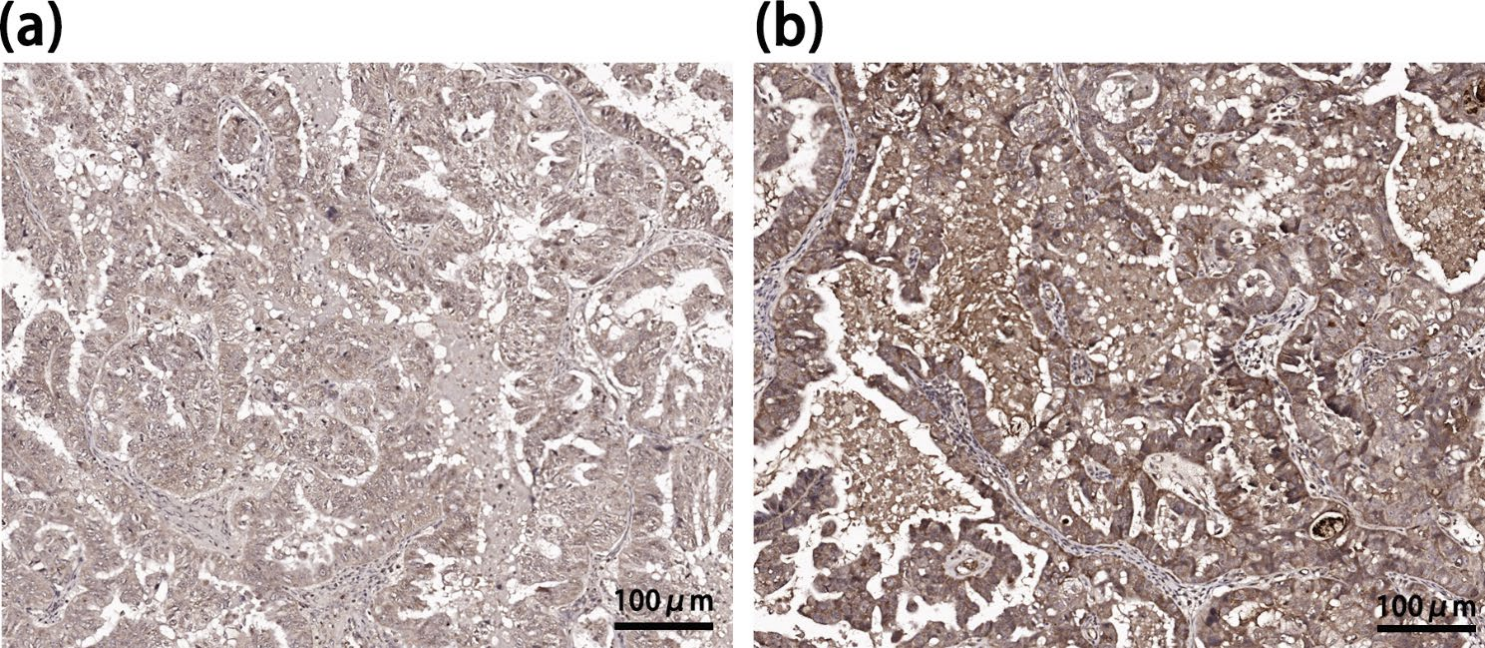
Paraffin sections of the patient’s ovarian tumor showed mild expression of **(a)** GluA1 (1:50), and **(b)** strong expression of GluA2 (1:50)

A 53-year-old woman with no previous history presented with short-term memory loss two days after a primary cytoreduction surgery (Ascites aspiration + surgical exploration + bilateral salpingo-oopherectomy + hysterectomy + omentectomy + para-aortic and pelvic lymph node dissection) for HGSOC (FIGO stage: IC). (Fig. 1) The anesthesia and surgical procedure went uncomplicated. No severe adverse event happened. The memory deficit did not affect the patient’s daily activities, and no progression was detected, so she was discharged ten days after the operation. Two weeks later, she was admitted to the neurology department due to confusion, disorientation, speech dysfunction, agitation, and hallucination. The physical examination showed short-term memory deficits, disorientation, and count disturbance. Chest and abdomen CT scans revealed negative results except for signs of mild pneumonia. The brain MRI and Magnetic resonance spectroscopy imaging (MRSI) revealed hippocampus atrophy and diminished NAA/(Cho + Cr) ratio in the bilateral hippocampus region (right 0.25, left 0.1), suggesting sclerosis and neuron damage. (Fig. 2) Cerebrospinal fluid (CSF) was notable for pleocytosis (309✕10^6^/mL) and elevated protein (0.688 g/L). The paraneoplastic antibody panel for CSF and serum returned positive for AMPAR antibody (CSF IgG 1:32; serum IgG 1:3.2, cell-based assay, Fig. S1), as well as anti-TRDNER antibody (+) and anti-Recoverin antibody (+), and the anti-AMPAR encephalitis was diagnosed. Moreover, the paraffin sections of the patient’s ovarian tumor demonstrated mild expression of AMPAR GluA1 and strong expression of GluA2. (Fig. 3) The patient was treated with Methylprednisolone sodium succinate (750 mg qd for seven days), intravenous immunoglobulin (IVIG) (15 g qd for five days), and acyclovir (0.5 g q8h for 15 days) for suspected limbic encephalitis and virus encephalitis.

A week after admission, the patient developed a vigil coma with ocular movement disorders, autonomic dysfunction of severe dysphoria, and epilepsy. The electroencephalography (EEG) was repeated twice without detecting epileptiform discharge.

**Fig. 4:**
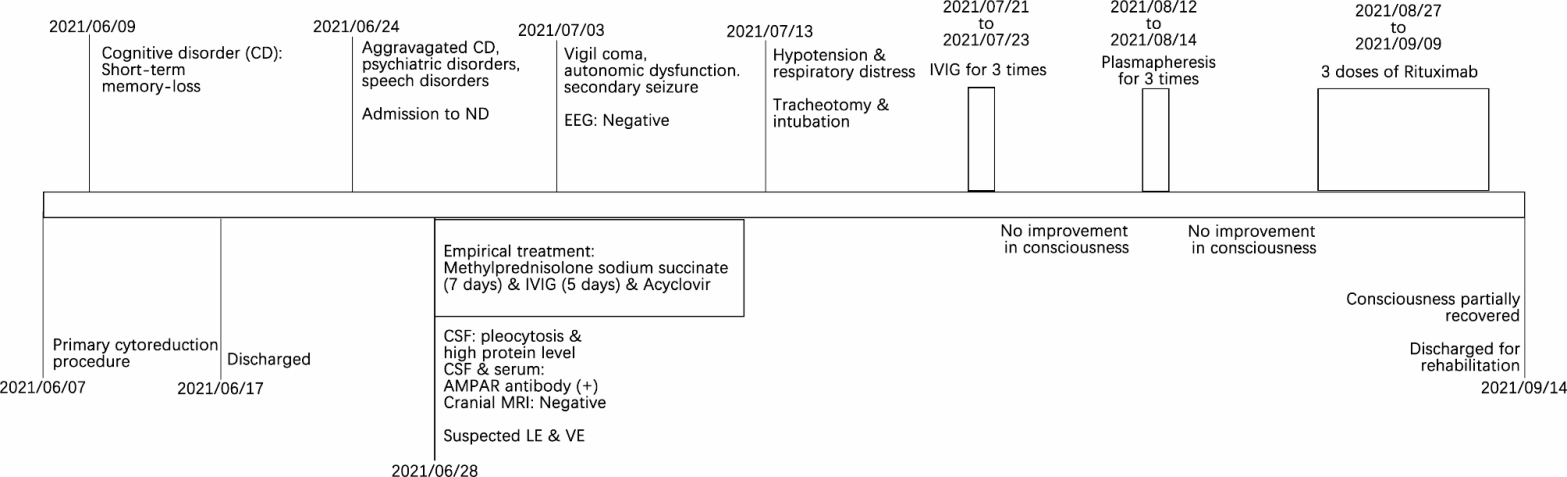
Timelines of clinical course and treatment of the patient with anti-AMPAR encephalitis associated with high grade serous ovarian cancer (HGSOC).

Nine days later, she developed hypotension and respiratory distress, leading to tracheotomy and intubation. Over the next month, she started another IVIG three times, followed by three cycles of plasmapheresis. The clinical improvement was limited. An FDG-PET/CT scan was performed, showing no signs of tumor. She was then treated with three doses of rituximab, improving her consciousness. She could respond to some simple physical examinations when she was discharged to a rehabilitation unit three months after admission. (Fig. 4)

In a recent follow-up, the patient reported full recovery of her consciousness, speech, and movement abilities, with mild short-term memory loss remaining. She has completed three cycles of chemotherapy. Physical examination, CT scan, and tumor biomarkers found no tumor recurrence.

## Discussion and conclusions

We retrieved previously reported cases and identified 66 cases of anti-AMPAR encephalitis associated with tumors [1, 4–15]. Among these cases, thymic tumors (n = 30, median age 45 year, including 16 patients with thymoma, 13 patients with thymic carcinoma, and one patient with thymic carcinoid) were the most common, followed by lung cancer (n = 17, median age 65 year, including 11 patients with small cell lung cancer, two patients with non-small cell lung cancer, and four patients with undefined lung cancer), ovarian tumors (n = 8, median age 42 year, including two patients with ovarian adenocarcinoma and six patients with teratoma), breast cancer (n = 7, median age 61 year), medullary thyroid cancer (n = 1, age 69 year), melanoma (n = 1, age 61 year), Ewing sarcoma (n = 1, age 19 year), and seminoma (n = 1, age 36 year). The clinical manifestations varied among different tumor types. (Table 1)

**Table 1:** Clinical characteristics, treatment, and outcomes in anti-AMPAR encephalitis patients with tumors

		Patients with thymic tumor (n = 30) (%)	Patients with lung cancer (n = 17) (%)	Patients with ovarian tumor (n = 8) (%)	Patients with breast cancer (n = 7) (%)	Patients with other types of cancer (n = 4) (%)
**Age (median ± SEM, yr) (n)**		**45 ± 3.2 (19)**	**65 ± 2.3**	**42 ± 8.9 (4)**	**61 ± 4.0 (6)**	**46 ± 11.5**
**Main encephalitis-related symptoms**	**Cognitive dysfunction**	**25 (83.3)**	**16 (94.1)**	**4/4 (100)**	**6/6 (100)**	**4 (100)**
	Memory deficits	19 (63.3)	16 (94.1)	3/4 (75)	6/6 (100)	4 (100)
	Disorientation	4 (13.3)	4 (23.5)	1/4 (25)	1/6 (16.7)	0
	**Psychiatric symptoms**	**22 (73.3)**	**11 (64.7)**	**4/4 (100)**	**5/6 (83.3)**	**1 (25)**
	Anxiety	0	1 (5.9)	1/4 (25)	0	0
	Depressed mood	4 (13.3)	2 (11.8)	1/4 (25)	0	0
	Abnormal behavior	15 (50)	9 (52.9)	1/4 (25)	4/6 (66.7)	1 (25)
	Sleep issues	3 (10)	4 (23.5)	0	1/6 (16.7)	0
	Psychotic-related symptoms	8 (26.7)	4 (23.5)	4/4 (100)	3/6 (50)	1 (25)
	Catatonia	1 (3.3)	0	0	0	0
	Dementia	1 (3.3)	0	0	0	0
	**Consciousness dysfunction**	**9 (30)**	**9 (52.9)**	**7 (87.5)**	**6/6 (100)**	**3 (75)**
	**Seizures**	**8 (26.7)**	**3 (17.6)**	**2/4 (50)**	**1/6 (16.7)**	**0**
	**Dyskinesia**	**5 (16.7)**	**2 (11.8)**	**2/4 (50)**	**1/6 (16.7)**	**0**
	Ataxia	0	2 (11.8)	0	1/6 (16.7)	0
	Dystonia	2 (6.7)	0	0	0	0
	Myoclonus	1 (3.3)	0	0	0	0
	Weakness	2 (6.7)	0	1/4 (25)	1/6 (16.7)	0
	Involuntary movement	1 (3.3)	0	1/4 (25)	0	0
	**Speech dysfunction**	**3 (10)**	**0**	**3/4 (60)**	**0**	**0**
	Verbal reduction	2 (6.7)	0	2/4 (50)	0	0
	Aphasia	2 (6.7)	0	1/4 (25)	0	0
	**Autonomic dysfunction**	**4 (13.3)**	**0**	**3/4 (75)**	**0**	**0**
	Respiratory distress	4 (13.3)	0	1 (25)	0	0
	**Others**	**6 (20)**	**2 (11.8)**	**0**	**0**	**1 (25)**
	Sensory dysfunction symptoms	1 (3.3)	2 (11.8)	0	0	0
	Headache	2 (6.7)	1 (5.9)	0	0	0
	Fever	5 (16.7)	0	0	0	0
	Hyponatremia	0	2 (11.8)	0	0	1 (25)
**Initial disease**	**Encephalitis**	16 (53.3)	14 (82.4)	2 (25)	5 (71.4)	3 (75)
	**Tumor**	3 (10)	2 (11.8)	2 (25)	2 (28.6)	1 (25)
	**NA**	11 (36.7)	1 (5.9)	4 (50)	0	0
**ICU admission**		6 (20)	0	6/7 (85.7)	0	1 (25)
**Treatment**	**Immunotherapy**					
	First-line	11 (36.7)	13 (76.5)	1 (12.5)	7 (100)	4 (100)
	First-line + second-line	5 (16.7)	1 (5.9)	7 (87.5)	0	0
	**Tumor treatment**					
	Tumor removal	6 (20)	2 (11.8)	6 (75)	1 (14.3)	0
	Chemotherapy	0	8 (47.1)	2 (25)	1 (14.3)	1 (25)
	Tumor removal + chemotherapy	1 (3.3)	0	0	1 (14.3)	1 (25)
	Tumor removal + radiotherapy	3 (10)	0	0	0	0
	Tumor removal + chemotherapy + radiotherapy	1 (3.3)	0	0	2 (28.6)	1 (25)
	Chemotherapy + radiotherapy	0	4 (23.5)	0	0	0
	**Symptomatic treatment**	0	1 (5.9)	0	0	0
	**NA**	11 (36.7)	0	0	0	0
**Outcome**	mRS ≤ 2	10 (33.3)	4 (23.5)	3 (37.5)	3 (42.9)	0
	Symptom partially improved	4 (13.3)	2 (11.8)	1 (12.5)	3 (42.9)	1 (25)
	Symptom not improved	0	3 (17.6)	0	0	3 (75)
	Relapse	4 (13.3)	0	0	0	0
	Dead	6 (20)	8 (47.1)	0	0	0
	NA	6 (20)	0	4 (50)	1 (14.3)	0

AMPAR antibody titers in the serum or CSF were reported in 14 patients (Table 2). Notably, the CSF titer of several patients was negative; thus, acquiring paired serum and CSF samples of patients suspected of anti-AMPAR encephalitis is essential [16, 17]. The AMPAR antibody would decrease after immunotherapy [3, 18–20]. In our reported case, however, we did not obtain the patient’s antibody titer after immunotherapy because her relatives refused lumbar puncture. Severe symptoms such as consciousness and autonomic dysfunction are thought to be related to higher titers of AE antibodies. However, from the previous cases, we observed some patients presenting mild symptoms but with high titers in serum and/or CSF and some patients being in fatal status but had relatively lower titers [17, 20–22]. We supposed that the inconsistency was due to the concurrent onconeural antibodies, and the tumor itself might also have an impact on the severity of symptoms.

**Table 2:** Anti-AMPAR antibody titer, clinical manifestations, ICU admission, and outcomes in anti-AMPAR encephalitis patients with tumors

Case	Tumor type	Anti-AMPAR antibody titer		Clinical Manifestations	ICU admission	Outcome
		Serum	CSF			
Ricken, 2021	Lung cancer	1:1600	1:32	Purely amnestic syndrome	Y	Died 3.5 mo after onset because of tumor progression
Yang, 2021	Lung cancer	+	1:10	short-term memory loss, abnormal psychological behaviors	NA	Died of respiratory failure
Zhang, 2021	Lung cancer	1:10	-	Amnesia, confusion, psychiatric disturbances	NA	Improvement in mood disorders and psychosis die of tumor
Zhang, 2021	Lung cancer	1:10	-	Psychiatric disturbances and amnesia	Y	mRS = 0
Dogan Onugoren, 2015	Ovarian tumor	1:16000	NA	Memory deficits (multi-phase), psychosyndrome	NA	mRS = 0
Presented case	Ovarian tumor	1:3.2	1:32	Cognitive dysfunction, consciousness and autonomic dysfunction	Y	mRS = 0
Bataller, 2010	Breast cancer	NA	1:10	Confusion, hypersomnia, visual hallucinations, and combativeness	NA	mRS = 0
LauridoSoto, 2019	Thymic tumor	1:256	1:256	Cognitive dysfunction, psychiatric symptoms, dyskinesia, hypoventilation	Y	mRS = 0
Yang, 2016	Thymic tumor	1:10	-	Paroxysmal paresthesia, seizure, consciousness dysfunction	NA	Died 2 months later
Zhang, 2021	Thymic tumor	1:100	-	Psychiatric disturbances, amnesia, confusion	NA	Died
Zhang, 2021	Thymic tumor	1:100	1:100	Amnesia, consciousness dysfunction, psychiatric disturbances, dyskinesia, right face and perioral numbness	NA	mRS = 0
Luo, 2019	Thymic tumor	1:1000	1:32	Cognitive dysfunction, psychiatric symptoms, autonomic dysfunction	NA	Mild long-time memory deficits
Safadi, 2020	Thymic tumor	NA	1:256 1:3.2(after immunotherapy)	Consciousness and autonomic dysfunction	Y	Significantly improved
Qiao, 2021	Thymic tumor	1:32 1:32(after immunotherapy)	1:3.2 1:1(after immunotherapy)	Cognitive dysfunction, consciousness dysfunction, psychiatric symptoms, dyskinesia	Y	Remained unable to identify his family members, and the aphasia persisted

Y, the patient had ICU admission; NA, data not available

For patients with paraneoplastic anti-AMPAR encephalitis, both immunotherapies and tumor treatment are warranted. Almost all patients (87.5%) diagnosed with anti-AMPAR encephalitis associated with ovarian tumors received second-line immunotherapy, exceeding other tumors. ICU admission was also more likely to be reported in patients with ovarian tumors (85.7%), indicating anti-AMPAR encephalitis associated with ovarian tumors could deteriorate rapidly, and patients are more likely to suffer from fatal situations such as severe autonomic dysfunction and consciousness dysfunction. Thus, the first-line immunotherapies did not bring significant effects (Table 1). Furthermore, we supposed that the limited clinical improvement was also due to the clinical course specificity of the patient. For autoimmune encephalitis patients treated in our hospital and in cases reported previously, we found that the course of the disease varied in patients; some had a course several years long [3, 4, 9, 23]. For patients with a long disease course, treatments such as IVIG and plasmapheresis could not improve their symptoms in the short phase. For this group of patients, a second-line agent was applied. Due to its low toxicity and direct effects on B cells, rituximab is more commonly prescribed as the second-line agent by clinicians to improve patients’ outcomes. Moreover, some clinicians believe that adding second-line immunotherapy can also prevent the early relapse of AE [1]. However, there is no consensus for the treatment of autoimmune encephalitis except pediatric NMDAR encephalitis [24]. A multi-institutional observational study found that for patients who had no improvements within four weeks with first-line therapy, those who received second-line therapy had better outcomes than those who received either continued first-line treatment or discontinued therapy [25]. The result could be explained by the opinion that the second-line therapy should be initiated if patients do not respond to the first-line therapy within 4 weeks. Due to the mechanisms of rituximab and cyclophosphamide, the above result could be interpreted that for this group of patients, rituximab and/or cyclophosphamide should be applied as the first-line therapy [26]. A recent meta-analysis found that therapeutic apheresis alone or combined first-line therapy (corticosteroids and IVIG; corticosteroids, IVIG, and therapeutic apheresis) indicated good outcomes, providing evidence for first-line therapy selection. The study also concluded that timely initiation of second-line therapy was associated with better outcomes [27]. Since anti-AMPAR encephalitis patients associated with ovarian tumors were more likely to be in a fatal state, it is urgent to identify this patient group and initiate immunotherapy and vital support as early as possible.

We report the first case of anti-AMPAR encephalitis developed in an HGSOC patient after cytoreduction surgery. Cytoreduction surgery followed by platinum-based chemotherapy represents the standard treatment for epithelial ovarian cancer (EOC). Recently, new therapeutic strategies have proved to be effective. Adding bevacizumab to standard first-line platinum-based chemotherapy and to second-line therapy has demonstrated better survival outcomes in EOC patients. Although bevacizumab showed promising data in feasibility and safety as a neoadjuvant agent, it warrants further investigation in terms of administration timing considering the complications [28]. Poly (ADP-ribose) polymerase (PARP) inhibitors (PARP inhibitors) are emerging as the first-line maintenance treatment after platinum-based chemotherapy in newly diagnosed and as a maintenance treatment for relapse patients with BRCA mutated or HRD-positive settings [29]. Cytoreduction surgery refers to the curative treatment for peritoneal carcinoma. Its procedures include thorough surgical exploration and total omentectomy, with peritonectomy and organ(s) resection if suspecting carcinomas involvement [30]. Considering some patients’ encephalitis initiation began after cytoreduction surgery, we suggest cytoreduction surgery might be a trigger for AE. The tumor is considered a trigger for AE, our case and others reported GluA1/GluA2 expressions on the tumor tissue [1]. Surgical procedure might make it easier for the antigens on tumor tissue enter into circulation system, inducing the AMPAR antibodies production and encephalitis initiation. However, it is still unclear whether cytoreduction surgery or even tumor resection is related to the initiation of AE [1, 15, 31].

Of the previously reported anti-AMPAR encephalitis associated with ovarian tumors, one patient developed cognitive deficits following the surgery for ovarian adenocarcinoma [6, 9, 32]. Notably, we identified three cases in which patients developed encephalitis symptoms only a few days after tumor resection: two patients with breast cancer and another with thymoma [14, 15, 33]. Generally, patients with paraneoplastic AE develop encephalitis before detecting concurrent tumors or suggesting recurrent tumors [34–39]. However, some patients developed acute or subacute encephalitis symptoms after tumor resection [13–15, 33, 40]. For this group of patients, symptoms such as short-term memory loss and behavior change would develop in the non-neurology department and are easily neglected. Patients were discharged and readmitted in several weeks in our case and previously reported ones. If the possibility of AE could be suspected earlier, the earlier immunotherapy initiation might improve their outcomes. As a result, clinicians should consider AE as a differentiation diagnosis when postoperative patients with tumors develop acute/subacute symptoms indicating multifocal brain inflammation.

Here, we present an approach to detecting probable AE patients in the gynecology department. (Fig. S2) Perioperative diseases (including pulmonary embolism, ischemic stroke, and electrocyte abnormalities), neurological diseases (including cranial hemorrhage, infectious encephalitis, Hashimoto encephalitis, and brain metastases related to ovarian cancer), and postoperative delirium and cognitive dysfunction should be considered as differential diagnosis [41–45].

Anti-AMPAR encephalitis is rare, with various acute or subacute onset symptoms, including cognitive deficits, psychosis, and decreased consciousness. Most patients can respond to immunotherapy. Some of them, however, might experience life-threatening occasions. As a result, early diagnosis and quick initiation of immunotherapy, and mature basic life support techniques are required. The disease might develop postoperatively if the tumor is a trigger of AE, and cytoreduction surgery might further stimulate the antigens into the circulation system; thus, surgeons should keep alarmed at patients’ cognitive and consciousness conditions, especially those who undertake surgeries for breast cancer, thymoma, or ovarian cancer.

### Electronic supplementary material

Below is the link to the electronic supplementary material.

Supplementary Material 1

## Data Availability

Not applicable.

## List of Abbreviations

AE: Autoimmune encephalitis
AMPAR: Anti-Alpha-Amino-3-Hydroxy-5-Methyl-4-Isoxazolepropionic acid receptor
CSF: Cerebrospinal fluid
HGSOC: High-grade serous ovarian cancer
IVIG: Intravenous immunoglobulin
MRSI: Magnetic resonance spectroscopy imaging
