# Supplementary material for: Anti-alpha-amino-3-hydroxy-5-methyl-4-isoxazolepropionic acid receptor encephalitis developed after ovarian cancer cytoreduction surgery: a case report and literature review
Source: BMC Womens Health. 2023 Sep 21;23:507. doi: 10.1186/s12905-023-02636-1 (PMC10512534; doi:10.1186/s12905-023-02636-1)
Supplement: Supplementary file 1 — Supplementary Material 1 [file 12905_2023_2636_MOESM1_ESM.pdf]

Appendix

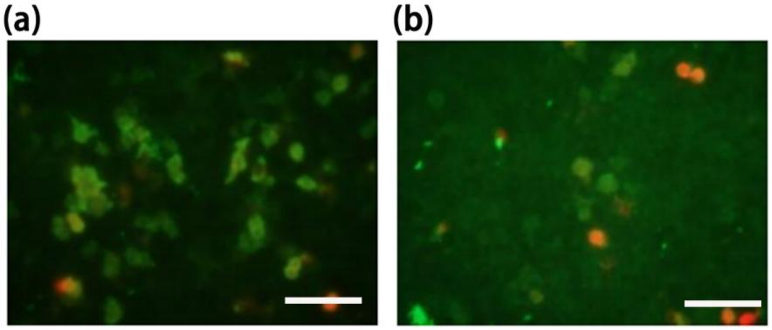

Fig. S1 HEK293 cells expressing the AMPAR (GluR2) reacted with the CSF and serum. The titer of antibodies in (a) CSF was measured 1:32 and in (b) serum was measured 1:3.2 (original magnification ×200, scale bar 50μm, provided by Chengdu Heimeryunying Center for Clinical Laboratory).

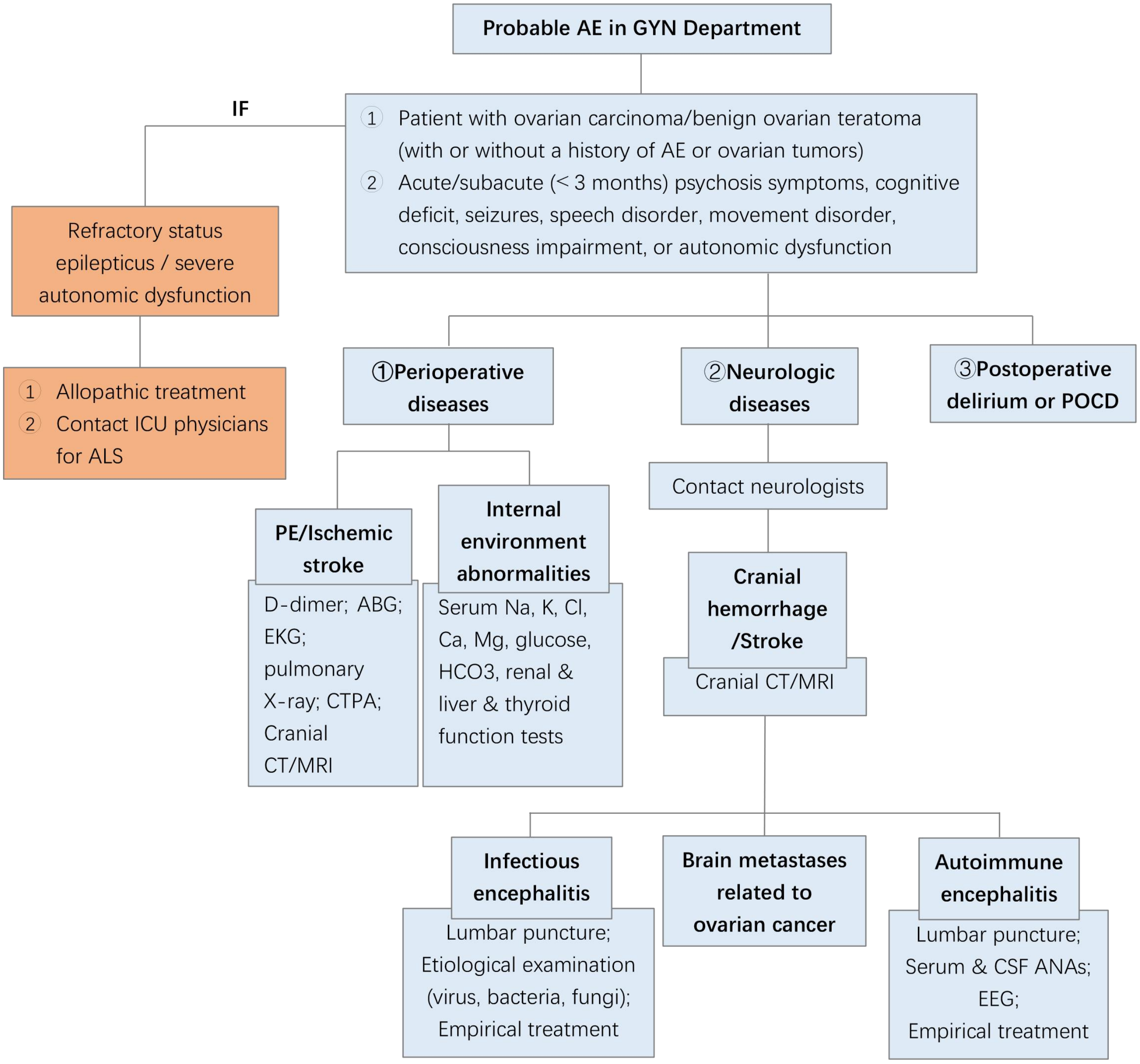

Fig. S2. An approach to detecting probable autoimmune encephalitis patients in the gynecologic department.
